# TGF-*β*1/Smads and miR-21 in Renal Fibrosis and Inflammation

**DOI:** 10.1155/2016/8319283

**Published:** 2016-08-17

**Authors:** Agnieszka Loboda, Mateusz Sobczak, Alicja Jozkowicz, Jozef Dulak

**Affiliations:** Department of Medical Biotechnology, Faculty of Biochemistry, Biophysics and Biotechnology, Jagiellonian University, 30-387 Kraków, Poland

## Abstract

Renal fibrosis, irrespective of its etiology, is a final common stage of almost all chronic kidney diseases. Increased apoptosis, epithelial-to-mesenchymal transition, and inflammatory cell infiltration characterize the injured kidney. On the molecular level, transforming growth factor-*β*1 (TGF-*β*1)-Smad3 signaling pathway plays a central role in fibrotic kidney disease. Recent findings indicate the prominent role of microRNAs, small noncoding RNA molecules that inhibit gene expression through the posttranscriptional repression of their target mRNAs, in different pathologic conditions, including renal pathophysiology. miR-21 was also shown to play a dynamic role in inflammatory responses and in accelerating injury responses to promote organ failure and fibrosis. Understanding the cellular and molecular bases of miR-21 involvement in the pathogenesis of kidney diseases, including inflammatory reaction, could be crucial for their early diagnosis. Moreover, the possibility of influencing miR-21 level by specific antagomirs may be considered as an approach for treatment of renal diseases.

## 1. Introduction

**Figure 1:**
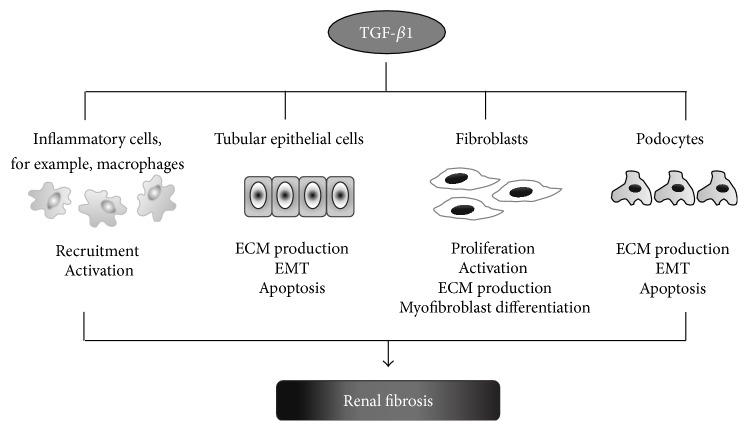
Role of TGF-*β*1 in kidney. Multiple effects exerted by TGF-*β*1 on various cells: podocytes, tubular epithelial cells, and inflammatory cells, for example macrophages, leading to their apoptosis, increased extracellular matrix (ECM) production, epithelial-to-mesenchymal transition (EMT), or activation.

The importance of fibrotic diseases rises in a global awareness, as approximately 45% of all deaths in the Western world are related to various forms of fibrosis [1]. The epidemiologic studies show that the number of patients with end-stage kidney disease is increasing worldwide. Renal fibrosis, irrespective of its etiology, is a final common stage of almost all chronic kidney diseases. Fibrosis develops in response to injury, when the normal wound-healing process is dysregulated and pathologically sustained. Excessive deposition of extracellular matrix (ECM) is a hallmark of all fibrotic diseases as ECM accumulation replaces functional tissue with a scar and this process alters organ physiological function and leads to its failure [2]. The majority of studies assign a key role in fibrotic progression to transforming growth factor-*β* (TGF-*β*), which executes its biological function by downstream activation of Smad signaling pathway [3]. TGF-*β*1, the most abundant isoform of TGF-*β* family members, can be secreted by all types of renal cells and infiltrated inflammatory cells. In turn, this cytokine acts on many types of cells present in kidneys, not only podocytes or tubular epithelia cells but also on inflammatory cells, including macrophages or T cells (Figure 1). A link between renal inflammation and fibrosis is well established and contribution of leukocytes to inflammation-driven fibrosis is stressed on (reviewed in [4]).

Recent years have also brought an enormous amount of data pointing to the role of microRNAs in pathologic conditions, including renal fibrosis, where changes in the expression of mostly miR-21, miR-29, miR-192, and miR-200 have been described [5]. Given the findings from the recent years that emphasize its significance in chronic kidney diseases as well as in inflammatory reaction, this review will focus on miR-21 and its therapeutic potential, as well as on its key upstream regulator—Smad3.

## 2. TGF-***β***: A Key Signaling Pathway in Organ Fibrosis and Inflammation

The TGF-*β* superfamily of growth factors is essential for the embryonic development and tissue homeostasis [6]. Over 30 different polypeptides, including contradictory TGF-*β*s and bone morphogenetic proteins (BMPs), secreted by different types of cells, share a set of common sequences and structural features.

The first step in TGF-*β* signaling requires receptor activation. Two different transmembrane, heterodimeric receptors, type I and type II, with serine/threonine kinases activity are required for TGF-*β*1 signal transmission. Noteworthily, the ligand, TGF-*β*1, is synthesized as latent form in association with latency-associated peptide (LAP) and binds to latent TGF-*β*-binding protein (LTBP) in the target tissues. It could be easily activated by various stimuli including reactive oxygen species (ROS) or plasmin and then can be released from the LAP and LTBP and becomes active [7]. The existence of different forms of TGF-*β* increases the complexity of the cellular response (see below). The active TGF-*β* ligand binds to receptor II and initiates phosphorylation and activation of receptor I, which in turn phosphorylates and activates recruited Smad proteins, what constitutes the second step in TGF-*β*1 signaling [8]. Worth mentioning, little is known about the mechanism underlying TGF-*β*/Smad interaction. Wei et al. suggested that the adaptor protein Kindlin-2 physically interacts with both TGF*β*RI and Smad3 and represents a link between TGF-*β* and Smad proteins and therefore it contributes to fibrosis [9].

TGF-*β* activation regulates diverse cellular functions including proliferation, apoptosis, differentiation, and inflammation [10, 11]. Many studies indicate a central role of TGF-*β* in the pathogenesis of renal fibrosis [12]. TGF-*β*1 exerts its profibrotic activity through stimulation of fibroblast proliferation, extracellular matrix synthesis (e.g., collagen types I, III, and IV, proteoglycans, laminin, and fibronectin), and epithelial-to-mesenchymal transition (EMT). Induced expression of ECM remodeling genes, increase in the apoptosis rate, and EMT lead to tubulointerstitial fibrosis and glomerulosclerosis (Figure 1). Distinct morphological changes during EMT are caused among others by TGF-*β*1-mediated downregulation of E-cadherin and upregulation of N-cadherin and vimentin. The switch in cell differentiation and behavior is mediated by key transcription factors, like SNAIL, TWIST, and zinc-finger E-box-binding (ZEB) (reviewed in [13]). Importantly, TGF-*β*-induced EMT involves also the epigenetic modifications, including decrease of the heterochromatin mark H3-lys9 dimethylation (H3K9Me2) and increase of the euchromatin mark H3-lys4 trimethylation (H3K4Me3) in lysine-specific demethylase-1 (LSD1) dependent manner [14].

TGF-*β*1 is well known for its profibrotic activity during renal fibrosis; however its contribution to renal inflammation seems to be more complex. A number of* in vitro* and* in vivo* studies show proinflammatory effects of TGF-*β*1. For example, in human kidney proximal tubule cells, stimulation with 2 ng/mL TGF-*β*1 for 72 h led to increased production of inflammatory proteins (macrophage migration inhibitory factor, MIF, and monocyte chemoattractant protein-1, MCP-1) and this effect was downregulated by overexpression of KLF4 (Krüppel-like factor 4), factor known to inhibit inflammation [15]. Bettelli et al. demonstrated that TGF-*β*1 (in cooperation with IL-6) favors the differentiation of proinflammatory T helper 17 (Th17) cells from naive T cells [16].

On the other hand, knockout of TGF-*β*1 leads to massive inflammatory disease in many tissues with the heart and lungs mostly affected [17, 18]. Moreover, the overexpression of latent TGF-*β*1 exerts protective effect in several models of renal injury. It was demonstrated that mice overexpressing latent TGF-*β*1 in keratinocytes were protected against renal fibrosis in a model of obstructive kidney disease [19]. In such model, severe renal inflammation, including massive T cell and macrophage infiltration and marked upregulation of interleukin-1*β* (IL-1*β*), tumor necrosis factor-*α* (TNF-*α*), and intercellular adhesion molecule-1 (ICAM-1) were observed whereas these changes were prevented in transgenic animals. The mechanism of anti-inflammatory effect of TGF-*β*1 was associated with a significant upregulation of I*κ*B*α* and a suppression of NF-*κ*B activation in the diseased kidney [19]. Moreover, the expression of Smad7, an inhibitory Smad, was upregulated [17, 19]. Similarly, in a model of crescentic glomerulonephritis, TGF-*β*1 overexpression leads to a 70% decrease in the accumulation of T cells and macrophages and reduced expression of renal IL-1*β*, TNF-*α*, and MCP-1 by 70 to 80% in comparison to wild-type animals [15].* In vitro* study, performed on human kidney tubular epithelial cells (HKC-8), showed that one of the possible mechanisms of anti-inflammatory effect of TGF-*β*1 relies on the inhibition of RANTES expression through *β*-catenin-triggered blockade of NF-*κ*B signaling [20]. Increased expression of RANTES, CC-chemokine ligand 5 (CCL5), has been reported in various kidney disorders and its inhibition might be an important mechanism for treatment of acute kidney injury, renal transplant rejection, and chronic kidney insufficiency.

The discrepancies observed between the above-mentioned studies and opposite pro- and anti-inflammatory effects of TGF-*β*1 confirm the pleiotropic activity of this cytokine. The differences in this activity might be caused by the local microenvironment (e.g., presence of IL-6), by the heterogeneity of the cell types used, or by the fact that both latent and active forms of TGF-*β*1 were used in the experiments. As TGF-*β* activation from latency is controlled through several factors, including proteases such as plasmin and MMP-9, in spatial and temporal manner [21], the effects exerted by latent and active forms may involve distinct mechanisms. The exact molecular pathways regulated by active and latent TGF-*β*1 in renal inflammation remains largely unknown and more detailed experiments are needed. The one possible explanation might be the fact that latent TGF-*β*1 may bind its own receptor, glycoprotein A repetitions predominant protein (GARP), to exert its anti-inflammatory effects [22] and GARP itself was shown to exert anti-inflammatory effect [23]. Moreover, the mechanism of the upregulation of Smad7 by latent form of TGF-*β*1 [17, 19] and its involvement in the protection against inflammation has to be further studied in detail.

## 3. Smad Signaling Pathway

**Figure 2:**
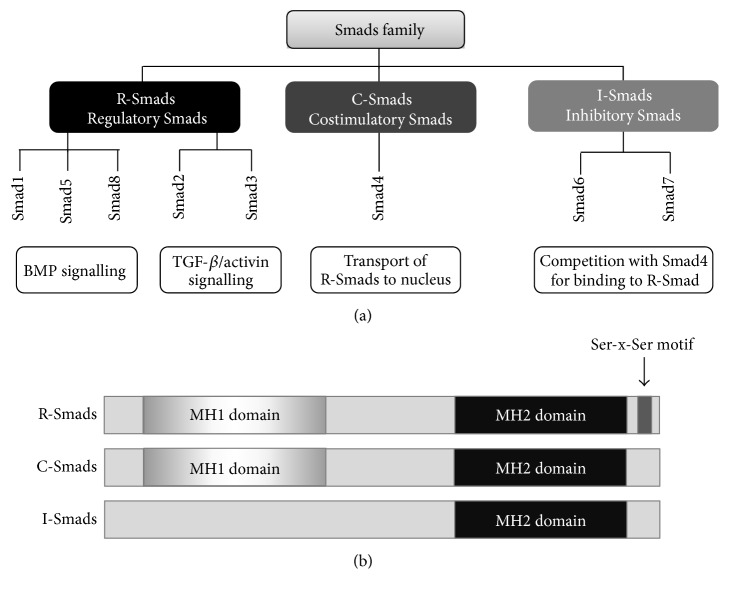
Smad family. (a) The family is composed of three groups: regulatory Smads or receptor-regulated Smads (R-Smads), costimulatory or common-partner Smads (C-Smads), and inhibitory Smads (I-Smads). R-Smads are the ligands for BMP, TGF-*β*, and activin receptors. Co-Smads are responsible for transport of R-Smads to nucleus, whereas I-Smads are negative regulators. (b) The members of each group are characterized by specific domains. R-Smad contains phosphorylation motif, Ser-x-Ser, at the c-terminal region. MH2 domain is present in all members, whereas MH1 domain is not present in I-Smads.

Smad transcription factors stand at the center of TGF-*β* signaling pathway. The name Smad originates from the combination of the names of two proteins—the MAD protein (mothers against decapentaplegic) that was found to mediate embryologic patterning in* Drosophila* and SMA proteins that were described in* C. elegans* [24, 25]. The proteins of a Smad family are present in nematodes, insects, and vertebrates and eight Smad proteins are found in the human and mice genomes [26]. The Smads are divided into three categories (Figure 2(a)). Five mammalian Smads, Smad1, Smad2, Smad3, Smad5, and Smad8, are named “receptor-regulated Smads” or “regulatory Smads” (R-Smads) and comprise substrates for BMP, TGF-*β*, and activin family of receptors. Receptor activated Smads are released from the receptor to form heterotrimeric complex together with a Smad4, a common-partner Smad—costimulatory Smad (C-Smad) for all R-Smads. A third category contains two members of the Smad family, Smad6 and Smad7, that act as inhibitory Smads (I-Smads), interfering with Smad-Smad or Smad-receptor interactions [11]. Of note, this system requires additional, cell-type-specific partner proteins in order to specifically transmit the signal in a context-dependent manner [27].

## 4. TGF-***β***/Smad Mode of Action

TGF-*β* stimulus has immediate effect on the expression of several hundred genes [28]. The same group of Smad proteins affects diverse gene expression patterns; therefore the result of TGF-*β* stimulation is cell- and context-dependent [10].

Receptor mediated induction of R-Smads takes place through direct phosphorylation of the two serines (Ser-x-Ser motif), located at the c-terminal “Mad homology-2” (MH2) domain, and constitutes the first stage in the TGF-*β* signaling pathway activation. MH2 is conserved domain shared among all Smad proteins and is responsible for Smad-TGF-*β* receptor interactions, transport into nucleus, and interactions with cofactors. In contrast, MH1 domain is not present in I-Smads (Figure 2(b)) [11]. In the next step of the signal transition, R-Smads, together with Smad4 (which lacks Ser-x-Ser motif; therefore it is not subjected to phosphorylation), form an oligomeric complex which is subsequently transported into the nucleus [11]. This multiprotein R-Smad-Smad4 assembly has the capacity to bind DNA through *β*-hairpin structure located at the N-terminal MH1 domain. In the major groove of the DNA, it forms hydrogen bonds with nucleotides within the specific SMAD binding element (SBE) that contains 5′-CAGAC-3′ nucleotide sequence [29]. Noteworthy Smad2 alone lacks this ability [11, 29]. The high specificity and affinity is achieved by the recruitment of other DNA-sequence-specific transcription factors, coactivators, and corepressors. Therefore, the proper transcriptional response depends on the particular set of additional partners that join Smad complex [30].

## 5. Diverse Roles of Smads in Renal Fibrosis and Inflammation

Activation of TGF-*β* pathway (largely driven by TGF-*β*1 isoform) in fibrosis and potent upregulation of Smad2 and Smad3 proteins have been demonstrated both in human and in animal models of chronic kidney disease [3].

**Figure 3:**
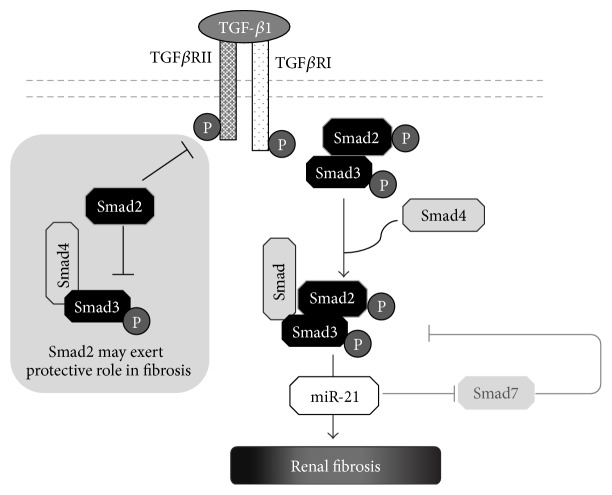
The role of Smad proteins in renal fibrosis. After binding to its receptor, TGF*β*1 stimulates Smad-mediated renal fibrosis. The phosphorylated Smad2 and Smad3 bind to Smad4 and form the Smad complex leading to the upregulation of miR-21 and fibrosis development. Of note, miR-21 inhibits Smad7, which under normal conditions acts as a negative regulator of TGF-*β*1/Smad3 signaling. Smad2 may protect against renal fibrosis through inhibition of Smad3 binding to TGF*β*RI as well as by blocking Smad3 nuclear translocation.

Importantly, both Smad2 and Smad3 proteins are phosphorylated in response to TGF-*β* receptor activation and in subsequent events become downstream mediators of TGF-*β* signaling. Phosphorylated Smad2 and Smad3 bind to the common Smad4 and form the Smad complex, which translocates into the nucleus to regulate the target gene transcription, including Smad7 (Figure 3).

The vast amount of data indicates Smad3 pathogenic role in fibrosis development. ECM deposition is a hallmark of all fibrotic diseases and, importantly, the expression of matrix proteins can be directly driven by Smad3 through its binding to specific promoter regions of collagen genes [31, 32]. Profibrotic role of Smad3 has been further demonstrated in knockout animal studies. Sato et al. showed that Smad3 is a critical factor for TGF-*β* effects in unilateral ureteral obstruction (UUO) model of renal fibrosis as Smad3 KO mice were protected against tubulointerstitial fibrosis [33]. Different studies, irrespective of applied model of kidney disease, pointed out Smad3 pathway as the central to the pathogenesis of interstitial fibrosis [34–36]. However, the complex interaction of a number of different cell types in injured kidney, both resident cells like tubular and endothelial cells as well as the inflammatory infiltrating cells like monocytes, macrophages, or T lymphocytes, may implicate the dynamic and diverse effects of Smad3 in various cell types in the kidney. Kellenberger et al. using Smad3−/− animals among others observed that the regulation of collagen *α*3(IV) gene expression is Smad3-dependent in glomerular endothelial cells whereas the regulation of MMP-2 gene expression is Smad3-dependent in mesangial cells and endothelial cells, but those factors are regulated in Smad3-independent way in the whole kidney tissue [37]. With regard to regulation of inflammatory response, Smad3 was shown to be critical for chemotaxis of macrophages [38], neutrophils [39], or transition of bone-marrow-derived macrophages into myofibroblasts [40]. In UUO model, in Smad3−/− mice, the inflammation was reduced as shown by hematoxylin-eosin staining as well as by the decreased number of F4/80-positive interstitial macrophages, CD4-positive T cells, and CD8-positive T cells [41].

Despite having very high (90%) structural similarity, Smad2 and Smad3 differ in functionality and they play opposing roles in renal fibrosis (Figure 3). Smad3 null mice die between 1 and 8 months due to immune defects [39]. On the other hand, mice lacking Smad2 show embryonic lethality, which indicates the importance of Smad2 in early development and therefore makes the functional studies of Smad2 in fibrogenesis difficult [42]. However, using Smad2 conditional knockout mice, Meng et al. demonstrated the protective role of this factor in renal fibrosis [43]. Mice conditionally deprived of Smad2 and subjected to UUO developed more severe tubulointerstitial fibrosis and similar results were obtained* in vitro* on cells stimulated with TGF-*β*1 [43]. It was suggested that Smad2 may inhibit Smad3 by opposing its phosphorylation and translocation into the nucleus and transcriptional activity (Figure 3, grey box). Unsurprisingly, Smad2 overexpression in tubular epithelial cells significantly reduced TGF-*β*1-induced phosphorylation of Smad3 protein and altered ECM proteins expression and turnover [43].

Similarly to Smad2, also Smad7, a member of inhibitory group of I-Smads, is a negative regulator of TGF-*β*1/Smad3 signaling, and a growing number of papers indicate its protective role in renal fibrosis [44, 45]. It also exerts anti-inflammatory effects, through targeting I*κ*B*α* and inhibition of NF-*κ*B activation [19]. Overexpression of Smad7 significantly reduced renal inflammation by suppressing the release of inflammatory cytokines, adhesion molecules, macrophages, and T cells activation [45].

The member of C-Smads, Smad4, is involved in the regulation of fibrosis and inflammation. As Smad4 knockout is embryonically lethal [46], model of conditional Smad4 deletion in tubular epithelial cells can be used to assess its effect in renal fibrosis and inflammation. In such mice, increased F4/80+ macrophages and CD45+ leukocytes infiltration as well as upregulation of proinflammatory IL-1*β*, TNF-*α*, MCP-1, and ICAM-1 in the obstructed kidney and in IL-1*β*-stimulated peritoneal macrophages was demonstrated [47]. In opposite, deletion of Smad4 inhibits progressive renal fibrosis* in vitro* and* in vivo* [47].

Altogether, these results indicate the complex scenario for Smads in the regulation of renal fibrosis and inflammation, which might be even more complicated by the fact that different microRNAs, small noncoding RNAs with a great regulatory potential, might be involved in this regulation.

## 6. Smad3 and miR-21 Regulation

Since the discovery of microRNAs (miRNAs) in* C. elegans*, deregulated specific miRNAs have been linked to the majority of diseases, including fibrosis [48]. Most miRNAs originate from long precursor molecules—double-stranded stem-loop structure bearing primary-miRNAs (pri-miRNA). Two RNase III enzymes, Drosha and Dicer, collaborate in the stepwise processing of miRNAs leading to the formation of functional miRNA-induced silencing complex (miRISC) to repress mRNA translation or induce its deadenylation and degradation [49]. Additionally, noncanonical mechanisms of microRNAs biosynthesis (Dicer- or Drosha-independent) are elegantly described in recent reviews [50, 51].

**Figure 4:**
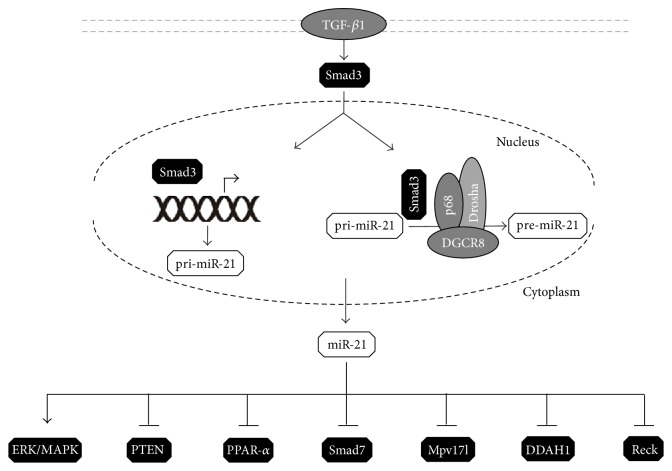
miR-21 regulation and mode of action. Upon activation, Smad3 is translocated into the nucleus, where it can regulate miR-21 expression. It can occur at the transcriptional level through binding to SBE located in the miR-21 promoter or posttranscriptionally through altering Drosha microprocessor complex, which results in increased processing of pri-miR-21 to pre-miR-21. miR-21 contributes to renal fibrosis by alteration of several metabolic pathways and targeting Smad7 protein, a negative regulator of TGF-*β*1/Smad3 signaling.

Importantly, the regulation of miRNAs expression through both transcriptional and posttranscriptional mechanisms might involve Smad proteins. Although Smads may regulate the expression of set miRNAs, especially Smad3 pathway changes expression of the key miRNAs involved in fibrosis. MH1 domain of Smad3 binds to Smad binding element (SBE) found in a variety of miRNA genes, including miR-21, miR-29, miR-192, and miR-200, what results in their up- or downregulation [52–55]. Recent studies have also demonstrated the role of Smad3 in stimulating miRNAs biogenesis [56–58]. In 2008, Davis et al. have shown that the induction of mature miR-21 and pre-miR-21 was not preceded by pri-miR-21 increase in response to TGF-*β*1-Smad3 stimulation [59]. This result indicated that the upregulation of miR-21 occurs at the posttranscriptional level; however, the exact mechanism of this regulation was not fully understood. Davis et al. (2008) suggested that Smad3, after translocation to the nucleus, associates with the Drosha/DGCR8/p68 microprocessor complex to facilitate the cleavage of pri-miRNA to pre-miRNA by Drosha [59]. In the later study, a group of miRNAs (including miR-21) posttranscriptionally regulated by TGF-*β* and BMP pathways was identified [58]. This regulation is enabled through direct binding of Smad proteins to the stem regions of the pri-miRNAs containing conserved sequence similar to the SBE sequence present in the promoters of Smad target genes. It has been suggested that Smad proteins are required for the efficient recruitment of the microprocessor complex to specific target pri-miRNAs. Later studies indicated that, regarding miR-21, Smad3 mediated posttranscriptional regulation plays a key role in its upregulation [55] (Figure 4).

## 7. miR-21 in Renal Fibrosis and Inflammation

From many microRNAs identified till now, miR-21 was shown to be upregulated in several distinct animal models of kidney disease and in both human acute kidney injury (AKI) and chronic kidney disease (CKD) tissue samples [60–62]. Of note, miR-21 is also one of the most highly expressed miRNAs in the healthy kidney [61, 63] and is expressed in many other uninjured organs [64]. However, under normal conditions (healthy tissues), miR-21 is rather nonfunctional and its activity is maintained below a threshold required for binding to target mRNAs and their silencing [65]. In the healthy kidneys, miR-21 is expressed mainly in the cortex. Following injury its expression is greatly increased and localized in a tubular epithelium. This suggests that miR-21 targets genes in tubular epithelial cells and mostly during injury [62].

Chau et al. managed to successfully generate miR-21 knockout mice [61]. The microarray comparison of healthy kidneys from miR-21 WT and KO mice showed no differences in the expression of genes predicted to be its targets, based on mRNA 3′UTR analysis, confirming that miR-21 is not important in normal tissue homoeostasis. The assumed changes appeared only after kidney injury following two most commonly used models of renal fibrosis—UUO and ischemia/reperfusion injury (IRI). This is in accordance with the observation by Androsavich et al. [65] who showed that the gain of miR-21 function in diseased or stressed cells is related to its enhanced association with polysome-associated mRNA and increased activity of miR-21 to bind target mRNAs than in normal conditions (healthy cells). Accordingly, Chau et al. observed that mice with functional miR-21 gene subjected to UUO or IRI developed more interstitial fibrosis, with higher apoptosis and myofibroblasts content [61].

The global analysis of gene expression profiles in miR-21 WT and KO mice in response to injury suggested that miR-21 affects fibrotic disease via regulation of metabolic pathways as most of the derepressed genes after miR-21 silencing were those involved in regulating fatty acid and lipid oxidation metabolic pathways [61]. Peroxisome proliferator-activated receptor-*α* (PPAR-*α*) is one of the most potent transcription factors that regulate fatty acid oxidation [66] and is predicted to be a major target for miR-21. It has been shown previously that miR-21 targets PPAR-*α* in endothelial cells [67]. In the normal kidneys, high expression of PPAR-*α* can be detected in the epithelium and interstitial cells, whereas, during injury, its expression is robustly decreased as a result of miR-21 targeting. In PPAR-*α* transgenic mice subjected to UUO reduced tubulointerstitial fibrosis, decreased ECM production and lower number of myofibroblasts in the interstitium were detected in comparison to WT mice [68]. Those observations stressed out the importance of fatty acids oxidation in kidney injury and fibrosis and directly points to miR-21-PPAR-*α* axis as a driving force of this alteration.

Generation of reactive oxygen species (ROS) contributes to cellular events that may result in fibrosis [69]. The mitochondrial inhibitor of ROS generation, Mpv17-like protein, has a high sequence homology with Mpv17 gene, a mitochondrial inner membrane protein that regulates the production of ROS and protects against mitochondrial oxidative stress and apoptosis [70]. Interestingly, kidneys of Mpv17 mutant mice developed progressive glomerulosclerosis [71]. Similarly to PPAR-*α*, Mpv17-like protein was found to be downregulated by miR-21 during kidney injury [61]. This resulted in a profibrotic ROS generation in the epithelium and outlines the role of miR-21 in this process.

Likewise in cardiac injury [72], miR-21 was also found to stimulate ERK/MAPK signaling in the kidney. This activation was significantly reduced in miR-21 KO mice and in mice injected with antagomir against miR-21 (anti-miR-21). However, in contrast to the heart fibroblasts, where this pathway was regulated through targeting Sprouty homologue 1 (Spry1) [72], in the kidney, despite miR-21 upregulation, this correlation was not found. This implicates for Spry1-independent mechanism of miR-21 activation of ERK/MAPK during kidney injury, probably through altered metabolic pathways [61].

The role of miR-21 in ECM homeostasis may be reflected by the regulation of MMPs and TIMPs expression. In fact, this relationship has been already well documented in cancers [73, 74]. Wang et al. [75] described the association between MMP-9/TIMP1 and miR-21 in renal fibrosis in diabetic nephropathy, confirming computational prediction pointing to MMP-9 as a potential target for miR-21 [61]. Additionally, this global transcriptomic analysis suggested another potent target of miR-21, involved in ECM regulation, namely, reversion-inducing-cysteine-rich protein with Kazal motifs (RECK), a metalloproteinase inhibitor [76]. Moreover, MMPs expression might be regulated by the tumor suppressor, the phosphatase and tensin homologue, known as PTEN. Chau et al. have shown that, in kidneys, miR-21 knockdown leads to MMP-2 decrease, which might suggest that PTEN is targeted by miR-21 also in the kidneys [61]. Through targeting PTEN and subsequent activation of Akt pathway, miR-21 may be involved in both EMT and endothelial-to-mesenchymal transition (EndMT) [77–80].

Yet another mechanism, where miR-21 plays a significant role, may contribute to loss of kidney function. As mentioned earlier, Smad7 is a negative regulator of TGF-*β*1/Smad3 signaling, which may reflect its protective role in renal fibrosis [44, 45] (Figure 3). Liu et al. predicted, using computational methods, that Smad7 is a miR-21 target in lung fibrosis and demonstrated that, indeed, Smad7 expression was negatively correlated with miR-21 [81]. This outlines the possible role of miR-21 in creating feedforward loop amplifying TGF-*β*1-Smad3 signaling. Similar correlation was observed by Zhong et al. in the study of renal injury in type 2 diabetes [82]. It was demonstrated that miR-21 overexpression or Smad7 knockdown ends with more severe renal fibrosis. On the other hand, miR-21 knockdown in diabetic kidneys resulted in restored Smad7 levels which was followed by reduced phosphorylation of Smad3 [82]. miR-21-dependent targeting PTEN and Smad7 was also found to be responsible for the progression of renal fibrosis in human diabetic nephropathy [83]. In a model of cyclosporine A- (CsA-) induced renal injury, downregulation of Smad7 and increase in TGF-*β*1 were independent of miR-21, but miR-21 mediated CsA nephrotoxicity via PTEN/Akt signaling pathway [84]. In patients with IgA nephropathy (IgAN) miR-21 was upregulated in both glomerular and tubular-interstitial tissues. Noteworthy, inhibition of miR-21 was able to prevent PTEN/Akt pathway activation and decrease fibrosis in podocytes and tubular cells [85].

Interesting finding was published recently by Liu et al. who suggested a new target for miR-21 action. In human HK-2 cell line, miR-21 interacts with 3′UTR of dimethylarginine dimethylaminohydrolase 1 (DDAH1), in Wnt dependent pathway [86]. DDAH1 is responsible for breakdown of asymmetric dimethylarginine (ADMA), an endogenous inhibitor of nitric oxide synthesis (NOS). Plasma ADMA accumulation, DDAH1 activity/expression reduction, and miR-21 upregulation might have the impact on renal fibrosis. It was already shown in 1996 that NO ameliorates UUO-induced fibrosis in rats [87] and inhibition of DDAH1 expression/activity resulting in NOS impairment might be responsible for complications observed in different renal diseases. During IRI, the reduction of DDAH1 expression and increased ADMA accumulation contributes to capillary loss and tubular necrosis in the kidney [88]. On the other hand, Zhao et al. demonstrated that DDAH1 reduction in mouse embryonic fibroblasts increases miR-21 expression and the susceptibility to oxidative stress [89]. Those data indicate the positive feedback regulation between miR-21 and DDAH1 and their relevance to kidney disease pathology.

In our hands, miR-21 was potently upregulated in a model of ochratoxin A- (OTA-) induced renal fibrosis. OTA is a common mycotoxin contaminating many food products and it has strong nephrotoxic activity [90]. OTA leads to induction of profibrotic TGF-*β* expression as well as the dysregulation of oxidant response [91, 92]. We have observed that miR-21 was one of the most highly induced miRNAs in OTA-treated animals (unpublished).

In summary, miR-21 contributes to renal fibrosis through multiple alterations in cell metabolism, the regulation of signaling pathways, like Akt and/or ERK/MAPK pathways and targeting Smad7 protein, a negative regulator of TGF-*β*1/Smad3 signaling (Figure 4). However, it has to be added that miR-21 may also act as a protective factor, for example, in glomerular injury. In patients with diabetic nephropathy, glomerular miR-21 was positively associated with albumin-to-creatinine ratio, whereas loss of miR-21 resulted in accelerated glomerular damage and podocyte apoptosis in a murine model of diabetic nephropathy and TGF-*β*1 transgenic mice [93].

miR-21 plays a dynamic role in inflammatory responses. It is induced in monocytic cells by LPS stimulation leading to inhibition of LPS-induced NF-*κ*B activation and IL-6 expression as well as to enhancement of IL-10 expression [1]. Targeting tumor suppressor programmed cell death protein 4 (PDCD4), a proinflammatory protein, might be the mechanisms of anti-inflammatory effect of miR-21 [94]. On the other hand, miR-21 through binding to receptors of the Toll-like receptor (TLR) family, TLR7 and TLR8 may also stimulate inflammation [95]. Such opposite effects exerted by miR-21 may explain why in the global analysis of gene expression in fibrotic kidney in miR-21 knockout animals the inflammatory pathway was not affected [61].

## 8. Therapeutic Potential and Biomarkers

So far, there is no effective therapy for renal fibrosis. Therefore, the focus needs to be put on designation of the new methods of treatment. From the therapeutic perspective, miR-21 seems to be very promising. miR-21 is regularly expressed in a healthy kidney; however it becomes active only after injury. Moreover, genetically modified mice with miR-21 silencing were as healthy as wild-type ones, but after kidney injury the global change in gene expression was observed [61]. It is very likely that this possible dormancy reduces the risk of any adverse effects when targeting miR-21. Furthermore, in the mouse models, administration of synthetic oligonucleotides which target complementary sequence in miR-21 greatly reduced kidney damage and fibrosis in mouse model of AKI [61], in the model of diabetic kidney disease [96], or in a chronic nephropathy known as Alport syndrome [63]. Similar observations were made with regard to heart and lung fibrosis, where, in each case, mice were subjected to miR-21 antisense oligonucleotides treatment [72, 81, 97]. Those findings raise the possibility of future clinical application. Importantly, in the above-mentioned studies, no deleterious effects of anti-miR-21 were described, although the compounds were given to animals for long time (e.g., several weeks [63]). Regulus Therapeutics is the example of the company focusing on targeting endogenous miRNAs, including miR-21, through inhibitory oligonucleotides [98]. The studies are underway, but more clinical trials with anti-miRNA are needed to fully address the safety issues in long-term delivery in humans. Currently, RG-012, the potent inhibitor of miR-21, is being evaluated in the Phase* *I* * clinical study to assess the safety, tolerability, and pharmacokinetics of subcutaneous dosing in healthy volunteers (ATHENA study). This has be followed by more clinical multicenter study to monitor the therapeutic effect of miR-21 inhibition on the decline in renal function and time to end-stage renal disease in patients.

There is a growing body of evidence that indicates the potent role of microRNAs in diagnostic field [99]. Indeed, in theory, these small RNA molecules possess many features of ideal biomarker: they are tissue- and disease-specific, stable in body fluids, and relatively easy to be quantified using already established methods [100]. With regard to kidney diseases, changes in the microRNAs that reflect kidney status can be detected in urine as it was previously shown for miR-29 [53], miR-93, and miR-21 [101]. Moreover, Mall et al. have shown that stability of miR-21 in human urine is relatively high [102]. Several harmful conditions have been tested, like long storage (5 days) at 4°C or subjection to ten freeze-thaw cycles at −80°C. The high stability of miR-21 supports its utility as urinary biomarker. However, data obtained by Wang et al. indicate that although miR-21 detected in the urine significantly correlated with glomerular filtration rate, it did not correlate with the extent of fibrosis in the histological analyses [101].

Glowacki et al. for the first time showed that increased serum levels of miR-21 may serve as a reliable marker that reflects fibrotic progression from its early stages [60]. Also recently serum miR-21 was suggested as a marker for diabetic nephropathy, and positive correlation between miR-21 and urine albumin creatinine ratio or content of collagen fibers has been demonstrated [103]. However, more studies are warranted to prove this finding and to give more information about miR-21 as a biomarker in renal fibrosis. One concern might be caused by the fact that miR-21 was suggested to be a general biomarker for different cancer and its upregulated level was detected in serum, plasma, and exosomes during tumor progression [104].

## 9. Conclusions

TGF-*β* pathway lies in the core of fibrotic changes in kidney. It executes its role by activating Smad3 protein, whose contribution in pathologic changes is well established and best demonstrated by the fact that Smad3 silencing leads to significant reduction of fibrosis. It has been shown that Smad3 not only regulates expression of various fibrotic genes but also influences miRNAs expression, both at the transcriptional and posttranscriptional level. Numerous reports point to deregulated miRNAs as a “driving force” of fibrosis. Among them miR-21 seems to have a particular impact on that process. Upregulation of the miR-21 expression alters metabolic pathways and leads to fibrosis. It was shown that in kidney epithelium miR-21 becomes active only in response to injury, which highlights its potential as a therapeutic target. This was confirmed also in animal models where silencing miR-21 either by gene knockout or by anti-miRs administration caused a significant fibrosis amelioration. Moreover, recent studies emphasize the possibility of employing circulating miR-21 as an early biomarker of renal fibrosis. Those results give the grounds for development of the new treatment strategies based on miR-21 inhibition and highlights the importance of gene silencing application in clinical trials.

## Abbreviations

ADMA: Asymmetric dimethylarginine
BMP: Bone morphogenetic proteins
CsA: Cyclosporine A
DDAH1: Dimethylarginine dimethylaminohydrolase 1
DGCR8: DiGeorge syndrome critical region gene 8
ECM: Extracellular matrix
ICAM-1: Intercellular adhesion molecule-1
IRI: Ischemia/reperfusion injury
KLF4: Krüppel-like factor 4
LAP: Latency-associated peptide
LSD1: Lysine-specific demethylase-1
LTBP: Latent TGF-*β*-binding protein
MCP-1: Monocyte chemoattractant protein-1
miRISC: MicroRNA-induced silencing complex
miRNA: MicroRNA
MMP-2: Matrix metalloproteinase-2
OTA: Ochratoxin A
Reck: Reversion-inducing-cysteine-rich protein with Kazal motifs
ROS: Reactive oxygen species
SBE: Binding element
Spry1: Sprouty homologue 1
TGF-*β*: Transforming growth factor-*β*
TIMP-1: Tissue inhibitor of metalloproteinase-1
UUO: Unilateral ureteral obstruction.
